# Cherokee Choices: A Diabetes Prevention Program for American Indians

**Published:** 2006-06-15

**Authors:** Jeffrey J Bachar, Lisa J Lefler, Lori Reed, Tara McCoy, Robin Bailey, Ronny Bell

**Affiliations:** Cherokee Choices/REACH 2010, Eastern Band of Cherokee Indians; Cherokee Choices/REACH 2010, Cherokee, NC; Cherokee Choices/REACH 2010, Cherokee, NC; Cherokee Choices/REACH 2010, Cherokee, NC; Cherokee Choices/REACH 2010, Cherokee, NC; Wake Forest University Health Sciences, Winston-Salem, NC

## Abstract

In 1999, the Centers for Disease Control and Prevention (CDC) provided Racial and Ethnic Approaches to Community Health 2010 (REACH 2010) funds to the Eastern Band of Cherokee Indians to develop a community-based intervention to improve the health of this rural, mountainous community in North Carolina. During the first year of the Cherokee Choices program, team members conducted formative research, formed coalitions, and developed a culturally appropriate community action plan for the prevention of type 2 diabetes, particularly among children. The Eastern Band of Cherokee Indians has higher rates of obesity and type 2 diabetes than the U.S. and North Carolina general populations. The Cherokee Choices program includes three main components: elementary school mentoring, worksite wellness for adults, and church-based health promotion. A social marketing strategy, including television advertisements and a television documentary series, supports the three components. School policy was altered to allow Cherokee Choices to have class time and after-school time devoted to health promotion activities. School staff have shown an interest in improving their health through attendance at fitness sessions. The credibility of the program has been validated through multiple invitations to participate in school events. Participants in the worksite wellness program have met dietary and physical activity goals, had reductions in body fat, and expressed enthusiasm for the program. A subcoalition has been formed to expand the worksite wellness component and link prevention efforts to health care cost reduction. Participants in the church program have walked more than 31,600 miles collectively.

## Health Issues Among the Eastern Band of Cherokee Indians

The Eastern Band of Cherokee Indians (EBCI) resides on the Qualla Boundary, which is nestled within the Great Smoky Mountains of western North Carolina. There are more than 13,000 enrolled members of the EBCI; about two thirds reside on tribal land in four counties. With the advent of casino revenues in 1997, economic changes have affected the EBCI. Within the last decade, the poverty rate fell from 31% to 22%, and the median family household income increased 82% to almost $32,000; this amount is still less than the state median by nearly $14,500 (1).

Although casino revenues have had a positive impact on family income, they may also have had a negative effect on family health behavior because families have more available funds to eat out. Sturm recently addressed trends in childhood obesity, including increases in soft drink consumption and carbohydrate intake, and noted that with increasing income, people are shifting toward eating food away from home because it is more convenient; these foods tend to be more energy-dense and contain more fats and sugars than foods prepared at home (2). Likewise, as family income has increased on the Cherokee Boundary (the primary Cherokee district), the array of fast food choices has increased; more than 19 fast food restaurants are available within 3 miles of the district's center.

Eighteen percent of 85 children surveyed in the EBCI community reported eating at fast food restaurants five or more times per week, and 52% reported eating out at least twice a week (3). Diet, along with sedentary lifestyle, may be contributing to the dramatic increase in childhood obesity among the EBCI. In 2003, 61.9% of EBCI boys aged 6 to 11 years who received services at the local hospital were overweight or obese, and 58.6% of EBCI girls in the same age group who received services at the local hospital were overweight or obese (4). In addition, children as young as 10 years have been diagnosed with adult onset diabetes (A. Bullock, MD, EBCI, oral communication, 2003).

**Figure 1:**
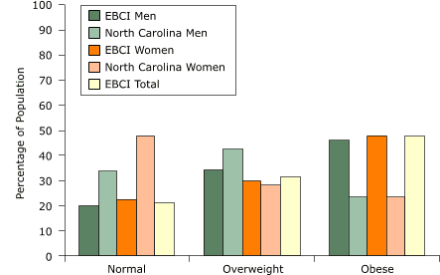
Rates of obesity and overweight among men and women of the Eastern Band of Cherokee Indians (EBCI) compared with men and women in North Carolina, March through June 2003 (6).

**Figure 2:**
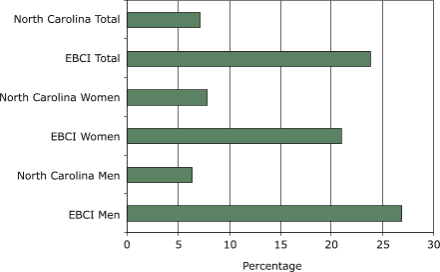
Rates of type 2 diabetes among men and women of the Eastern Band of Cherokee Indians (EBCI) compared with men and women in North Carolina, March through June 2003 (6).

The rates of obesity and type 2 diabetes among the EBCI exceed the rates for the U.S. and the North Carolina general population. In 2002, the age-adjusted prevalence of obesity among the U.S. population was 30% (5). According to data collected between March and June 2003, 45.7% of EBCI men and 23.5% of men in North Carolina were obese, and 47.9% of EBCI women and 23.6% of women in North Carolina were obese (Figure 1). Among EBCI, the prevalence of type 2 diabetes is 26.9% for men and 21.0% for women; the prevalence of type 2 diabetes is 6.4% among men in North Carolina and 7.9% among women (Figure 2). EBCI men and women reported a combined diabetes prevalence rate of 23.8%, more than three times the combined rate of 7.15% for men and women in North Carolina (6). In 2003, the prevalence of diabetes among U.S. men was 5.4%, and the prevalence among women was 4.7% (7).

**Table:** 

	**Normal**	**Overweight**	**Obese**
EBCI Men	20.1	34.2	45.7
NC Men	33.9	42.6	23.5
EBCI Women	22.6	29.6	47.9
NC Women	47.9	28.5	23.6
EBCI Total	21	31.4	47.6

**Table:** 

EBCI Men	26.9
North Carolina Men	6.4
EBCI Women	21
North Carolina Women	7.9
EBCI Total	23.8
North Carolina Total	7.15

In 1999, the Centers for Disease Control and Prevention (CDC) provided Racial and Ethnic Approaches to Community Health (REACH) 2010 funds to the EBCI to develop a community-based intervention to improve health. The REACH 2010 program is part of CDC's nationwide effort to reduce health disparities. An important role of the Cherokee Choices program among the EBCI is to listen to the Cherokee community and respond. The role involves being aware of and facilitating environmental, organizational, and individual changes. Priorities also include addressing racism, historic grief and trauma, mental health, and diabetes and obesity, and creating a supportive environment for developing positive policy changes. Diabetes and obesity were selected as the focus of the REACH 2010 program launched among the EBCI.

## Sociocultural Factors in Designing a Health Promotion Program

Many sociocultural factors were considered in designing and conducting a health promotion program among the EBCI. Although American Indian communities are not homogenous throughout North America, some traditional cultural values ring true for many. For example, many American Indians value the importance of spirituality for balance, the essential network of extended family systems, and the significance of intergenerational support. As part of the preparation for designing a culturally competent prevention program, it was understood that the Cherokee Choices team had to listen to the community and learn how to use these cultural values as strengths of the program. The team also needed to find out how people perceived the health issues that affected their families and community.

To address the two overarching health challenges of obesity and diabetes among the EBCI, Cherokee Choices used data from a CDC-initiated community health survey, formative research generated by Cherokee Choices program team members, and the guidance of a professional marketing agency. The marketing agency's initial report expressed concern about the indifference toward diabetes among the EBCI community:

Because diabetes has touched so many Cherokee families, there is a broad awareness of diabetes throughout the Cherokee community, accompanied by a general apathy. Because the disease is so rife, it has unfortunately created an almost fatalistic acceptance of diabetes as an "inevitable fact of Cherokee life" and a widespread belief that the disease is *not* preventable (The Goss Agency, written communication, 2004).

Medical anthropologists Singer and Clair (8) discuss the burden of coexisting diseases among populations and invoke the comments of Milstein (9) to exemplify the strategies used to address the excess burden of disease within a population: "To prevent a syndemic, one must not only prevent or control each disease but also the forces that tie those diseases together." One of the forces that had to be addressed initially by Cherokee Choices was the fatalism perceived by community members. To reverse this attitude, diabetes had to be brought to the forefront of the community. There had to be visual messages, mechanisms of emotional and educational support, and an approach that would create community coalitions with a single vision of making people healthier.

## Design and Implementation of Cherokee Choices

The Cherokee Choices/REACH 2010 program includes three main components: elementary school mentoring, worksite wellness for adults, and church-based health promotion. A social marketing component, including television advertisements and a television documentary series, supports the three components. The program is based on core American Indian values to maximize improvements to lifestyle behaviors and reduce risk of chronic disease.

During the first year of the program, team members conducted formative research, created coalitions, and developed an action plan. The formative research included in-depth interviews, a series of focus groups, and a review of epidemiological data. The focus groups collected information on the community's perceptions of diabetes and related health issues and their ideas and opinions for the program's design and initiatives. The research emphasized the prevention of type 2 diabetes, particularly among children. Recognizing that successful interventions for children need to include parents, the team identified the elementary school, worksites, and local churches as gateways for the intervention components. Evaluation of Cherokee Choices is based on the following REACH 2010 evaluation criteria: 1) targeted action, 2) community and systems change, and 3) change among change agents.

Meetings with tribal agencies and community groups facilitated planning and capacity building, which led to development of the community action plan. The plan embraced strategies to 1) engage individuals interested in a school intervention for children, 2) target tribal employees interested in losing weight and improving health, and 3) create opportunities for increased physical activity and nutritional information among church members.

### Elementary school mentoring program

This program was conducted at Cherokee Elementary School. The school is the one elementary school in the community, and it is operated by the EBCI. It includes grades kindergarten through six and has approximately 600 students. A team of four paid community mentors who met education and experience criteria worked with elementary school children and staff to increase awareness of diabetes as a serious health issue, promote physical activity and enhance knowledge about good nutrition during the school day and after school, teach stress-management techniques and coping skills, develop teachers as healthy role models through faculty fitness activities, and encourage healthy choices and general well-being with an overall objective to reduce the risk of diabetes. The mentors developed lesson plans that they implemented in the classroom to enhance self-esteem, cultural pride, conflict resolution, emotional well-being, and health knowledge. The mentors participated during class time, lunch, and recess. In addition, they developed a weekly after-school program to enhance teamwork and cultural awareness and increase physical activity.

The school component collected quantitative and qualitative data to contextualize responses and issues raised during the mentoring process. Students participated in an annual survey on issues of self-concept, perceived stress, cultural awareness, peer relations, and eating habits. Quantitative survey data were entered into SPSS 13.0 (SPSS Inc, Chicago, Ill) for analysis. The mentors also maintained daily logs in which they recorded interactions and exchanges between mentors and students. The narratives were entered into Atlas.ti (Atlas.ti Scientific Software Development GmbH, Berlin, Germany) a qualitative software program that systematically codes and catalogs for analyses. Mentors and program evaluation staff discussed patterns and themes that emerged from interviews to plan future program initiatives.

### Worksite wellness for adults

The worksite wellness program involved teams of tribal workers who were challenged to increase time spent in physical activity and participate in weekly educational and support activities. Tribal offices competed for prizes earned by attendance at healthy cooking demonstrations, classes on exercise techniques, nutritional assessments, supermarket tours, and stress-management workshops in addition to meeting physical activity and dietary change goals. Nutritionists, dietitians, and fitness workers conducted activities. Data were collected at baseline and follow up. Participants described their eating and physical activity habits through initial personal interviews conducted by Cherokee Choices program staff. Clinical measurements were taken at the local diabetes clinic. Measures included fasting blood glucose, blood pressure, and fasting lipid panel (total cholesterol, high-density lipoprotein, low-density lipoprotein, and triglycerides). Measurement of height, weight, and body fat percentage was recorded within a week of program enrollment. Body fat was measured with a Futrex–6100XL body-fat analyzer (Spencer Medical Inc, Rancho Santa Margarita, Calif), which can measure between 3% and 45% of body fat. Follow-up measures and interviews on goal attainment were conducted every 6 months. Data were entered into SPSS 13.0 (SPSS Inc, Chicago, Ill).

With this baseline information, services were developed and conducted at worksites. In addition, Cherokee Choices became active in health policy change with support from program managers, supervisors, and the EBCI chief, who attends many Cherokee Choices functions and celebrations.

The first group of worksite wellness participants began in June 2002 with two teams. During the next 2 years, seven more teams were formed for three additional 4-month challenges. More teams were added to waiting lists. A leader was selected for each team; the role of the team leader was to keep a record of weekly exercise minutes and participation in educational and support activities. Assessment data were entered into Access and ultimately translated into SPSS 12.0 (SPSS Inc, Chicago, Ill) for final analyses. Data collection methods include scheduled interviews, informal interviews, and client histories. Of 86 individuals who participated in the program for at least 1 year from June 2002 to June 2005, all but one continued to participate in the worksite program. Others joined the maintenance program for at least 1 year.

### Church wellness program

Nutritionists, dietitians, and fitness workers helped members of five churches participate in activities to improve diet and food preparation, raise awareness of tribal health-related services, and increase physical activity such as walking. The churches provided venues for healthy cooking demonstrations, exercise classes, and stress management lessons. Each church selected a team leader, and exercise classes were conducted in small group sessions. In addition, from November 2004 through April 2005, five churches participated in the Walk to Jerusalem, in which congregations organized walking groups and recorded their time and mileage walked. The goal of the program was to walk the equivalent distance from Cherokee, NC, to Jerusalem (approximately 8500 miles); progress was tracked on a map displayed prominently in each church. Each church member was given a pedometer to keep track of mileage, and incentives such as exercise videos, cookbooks, and weights were provided. Each participant filled out a preintervention and postintervention survey. Data included demographic information and queries on health, exercise time per week, and self-reports of daily water and fruit and vegetable intake.

## Social Marketing Initiative

We learned community concerns about the effects of diabetes through focus groups and individual interviews. People commented that participating in the focus groups gave them a new sense of camaraderie. The opportunity to share the experiences of a diagnosis of diabetes imparted the feeling that the participants were not alone. With this feedback, the program developed a seven-part television series of interviews with people who had experience with diabetes. Some interviewees had had diabetes for many years, others had been recently diagnosed, and still others did not have diabetes themselves but had diabetes in their families. Individuals shared how diabetes had affected their lives and how they had overcome the obstacles presented by the disease. The series was presented on the local cable channel. Anecdotal responses to the series included expressions of appreciation for airing it. In addition, Cherokee Choices/REACH 2010 collaborated with a marketing agency in developing three television spots that aired throughout western North Carolina. These 30-second videos depicted Cherokee community members engaged in healthy activities that resonated with themes generated by focus groups conducted by the marketing agency. The themes included family, spirituality, and tradition. The development of print advertising is in process.

## Consequences

Cherokee Choices/REACH 2010 has experienced success within each of the three program components.

### Elementary school mentoring program

Systems changes in the school have generated increased physical activity among students and staff, increased the fresh fruit and vegetable options in the school lunch menus, and increased parental participation in student activities.

Interviews with school staff illuminate the impact of Cherokee Choices/REACH 2010 on school culture. Changes include the following:

- The Cherokee Choices nutritionist is allowed to make changes in school menus. During summer school 2002, taste tests were conducted among students in grades kindergarten through 12 to determine potential modifications for healthier food selections.
- Teachers and staff have participated in fitness classes and workshops sponsored by Cherokee Choices since 2004.
- Teachers have been using a pamphlet developed by mentors in 2004 on healthy snacks for classroom parties.
- Sixth-grade teachers in 2004 opted to take the top reader awardees for a swim party to increase physical activity rather than a pizza party as in years past.
- During the annual school harvest festival, when parents traditionally have donated soft drinks to the school for prizes during the event, teachers and students urged parents to bring water, diet drinks, or flavored water instead.
- Cherokee Choices has a special display of "brain food" books that focus on health and diet. Children in grades four through six have been checking out these books frequently.
- An annual Cherokee Elementary School jump-rope competition and fundraiser for the American Heart Association became an annual Jump Against Diabetes event. Cherokee Choices was the first organization to assist the school when coaches and school administration decided to change the focus of the fundraiser. In 2005, more than $32,000 was raised locally for diabetes-related causes.
- Cherokee Elementary School staff and administration continue to work with Cherokee Choices on the mentoring program and related activities (e.g., Jump Against Diabetes, a spring art fair, the harvest festival, collection of data on the children's body mass index and weight, the after-school program).
- 96% of school program participants said they know how to make healthier food choices.

Results from psychosocial surveys of participating children have yielded promising results (Table 1). For example, children who spent time with mentors both during school and after school reported doing much better or a little better in the following categories: interest in school (71.8%), learning (82.1%), and ability to talk more easily with friends (66.7%). Children who spent time with mentors during school only reported doing much better or a little better in the same categories but at lower rates: interest in school (56.3%); learning (43.8%), and ability to talk more easily with friends (56.3%).

During interviews of school faculty and staff, one teacher with almost 30 years of experience at the school observed:

At Cherokee Elementary, I've noticed that since we've had Cherokee Choices in our school that the students are much more aware of the necessity of physical exercise, eating healthy, and making food choices. When they come to the library, they're looking for materials on cooking, they're looking for materials about their bodies and maturing, and we see them out walking on our track, and they're trying to be healthier overall.

She continued by saying,

I feel that Cherokee Choices has made a good impact on the community as far as educating the parents about how important it is that their child be eating healthy and making the right food decisions. We see lots more parents in the school being involved with activities that have been sponsored by Cherokee Choices. We also see Cherokee Choices at every event, and they're very cooperative and helpful when we have anything going on in the school.

Another teacher with more than 20 years of experience at the elementary school stated the following:

Being a Native American school, we have had a lot of groups and teams come in throughout the last years wanting to — quote — study the Native American students, and usually they left as fast as they came without any conclusions. We never saw the data, no suggestions, just basically what we were doing wrong. So naturally, when we met with Cherokee Choices a few years ago, there was a lot of skepticism. What exactly were they going to do different? How long were they going to stay? Are they going to help us or just tell us what we're doing wrong?  And I think what really piqued my interest was their philosophy of fighting diabetes. It's not just what you eat or exercise but how much stress and depression are contributive factors. They came into the school's culture to see what they were doing and actually became part of their lifestyle. They have been very supportive to students. We see them in the school regularly in the community in all the activities that we have. They're not just teaching them good nutrition, but they're teaching them how to make healthy lifestyle choices, how to work together, how to cooperate and support each other, how to express their feelings through artwork, and supporting each other, good teamwork, which is what Cherokees are supposed to do.

### Worksite wellness program

The percentage of people meeting physical activity recommendations, losing weight, and decreasing body fat increased among worksite wellness participants. In addition, attitudinal change among change agents contributed to support of worksite wellness activities. Tables 2 and 3 show that almost two thirds of participants lost weight and maintained weight loss and that one third lost one or more points in body mass index.

Program milestones include the following:

- There has been an increase in healthy eating behavior and physical activity reported by worksite wellness participants: 88% completed the program, 56% met goals, and 94% would participate again.
- Some participants have been able to decrease or eliminate diabetes medications, high blood pressure medications, or both.
- Employees have been given time off to exercise.
- Several abstracts have been submitted for publication, and three presentations have been made at annual meetings of the American Public Health Association.

### Church wellness program

The church component of Cherokee Choices has prompted pastors to develop a series of sermons that underscore the importance of taking care of the physical as well as the spiritual self. Participants in the Walk to Jerusalem walked more than 31,600 miles within 6 months. Each of the 150 participants walked an average 211 miles.

## Interpretation

The intervention has proven worthwhile on multiple levels. It serves as a model for developing future interventions locally. The community-based participatory approach of the intervention elicited high-quality community involvement and earned respect from community members. The approach adopted by Cherokee Choices is contrary to the history of top-down programs typically provided by social service agencies in Cherokee and has generated interest in using the same formative techniques for other health issues. The philosophy that underlies the Cherokee Choices intervention is that community and system changes can be effected through multiple, not necessarily linear, courses of action. It is essential to start with community members.

The success of participants in the worksite and church programs has inspired other worksites and churches to request an expansion of the Cherokee Choices program. Individuals have developed into role models who can help shift attitudes of coworkers, community members, and tribal leaders. There is a greater sense of hope among the community regarding diabetes prevention; however, there is still much work to be done.

We would advise others involved in funding and implementing similar work to allow an adequate amount of time for formative research. The use of a social network analysis is recommended as an initial step. In addition, we urge the use of social marketing in all phases of the intervention.

## Figures and Tables

**Table 1:** Comparison of Perceptions of Peer and School Relationships Among American Indian Children Aged 9 to 10 Years After 1 Year of the Cherokee Choices Intervention, North Carolina^a^

**Outcome**	**Mentoring and After-School Program N = 55**	**Mentoring Only N = 85**
**Since spending time with mentors, children who report "much better" or "a little better" this year, %**		
**School**		
Grades	71.8	68.8
Interest in school	71.8	56.3
**Peers**		
Friendships	59.0	62.5
Learning	82.1	43.8
Talk more easily with friends	66.7	56.3
Missed fewer days of school	61.5	62.5
Fewer conflicts	68.2	62.5

a *P* values were not calculated because of the sample size. The peer relationship survey was modified for this population from surveys suggested in the mentoring literature. Students were given five possible responses, varying in degree from "much better" to "a lot worse."

**Table 2:** Change in Weight and Body Mass Index (BMI) Among 86 Participants in the Cherokee Choices Worksite Wellness Program, June 2002 to June 2005, North Carolina

** Types of Change**	**No. (%)**
**Weight**	
Gained	23 (26.7)
No change	2 (2.3)
Lost	61 (70.9)
<5 lb	25 (29.1)
5 to <10 lb	11 (12.8)
10 to <20 lb	18 (20.9)
≥20 lb	7 (8.1)
**BMI**	
Increased	23 (27.0)
No change	2 (.02)
Decreased	61 (71)
.01 to <1.00 point	28 (32.6)
1.00 to 1.99 point	15 (17.4)
2.00 to 2.99 point	9 (10.5)
≥3.00 point	9 (10.5)

**Table 3:** Change in Mean Body Mass Index (BMI) Among 86 Participants in the Cherokee Choices Worksite Wellness Program, June 2002 to June 2005, North Carolina

** Sex**	**Starting BMI Mean (SD)**	**Final BMI Mean (SD)**	**Change**
Total	33.78 (7.00)	32.94 (7.06)	−0.85
Women	32.52 (6.73)	31.68 (7.12)	−0.84
Men	35.72 (7.07)	34.86 (6.60)	−0.85
